# Enhanced recovery after surgery compliance and postoperative recovery in elderly colorectal cancer patients: a propensity score matched cohort study

**DOI:** 10.3389/fonc.2026.1760602

**Published:** 2026-04-10

**Authors:** Juan Wang, Qiaolin Wang, Hailong Li, Jinlian Yu

**Affiliations:** 1 Department of Gastroenterology, Affiliated Hospital of Xuzhou Medical University, Xuzhou, Jiangsu, China; 2 Department of Surgery, Yantai Qishan Hospital, Yantai, Shandong, China; 3 Department of Blood Transfusion, Ulanqab Central Hospital, Ulanqab, Inner Mongolia, China; 4 Department of Gastroenterology, Bao Ji Central Hospital, Baoji, Shaanxi, China

**Keywords:** aged, colorectal neoplasms, enhanced recovery after surgery, length of stay, patient compliance

## Abstract

**Background:**

Enhanced Recovery After Surgery (ERAS) pathways are standard in colorectal surgery, but evidence in elderly patients is limited. This study evaluated the impact of ERAS adherence on outcomes after curative colorectal cancer (CRC) resection in patients ≥65 years.

**Methods:**

A single-centre, retrospective cohort of elderly CRC patients undergoing elective resection (2021–2024) was analysed. Adherence to 15 ERAS items was assessed; ≥80% (≥12 items) defined high compliance. Propensity score matching (1:1) balanced baseline characteristics. Primary endpoints were postoperative length of stay (LOS) and 30-day major morbidity; secondary endpoints included recovery milestones and 12-month functional outcomes.

**Results:**

Of 256 screened patients, 150 were matched (75 per group). High compliance was associated with shorter LOS, higher discharge within 10 days (73.3% vs 40.0%; p<0.01), and fewer major complications (14.0% vs 22.0%; p=0.043). Recovery milestones occurred earlier in the high-compliance group: ambulation (22.6 vs 26.1 h), bowel recovery (32.1 vs 36.0 h), and oral intake (30.5 vs 34.8 h). At 12 months, more patients resumed a normal diet without obstructive symptoms (92.9% vs 81.5%; p=0.021).

**Conclusions:**

In elderly CRC patients, high ERAS compliance was linked to shorter LOS, reduced morbidity, faster recovery, and sustained dietary benefit. Improving adherence may optimise outcomes, warranting validation in multicentre studies.

## 1 Introduction

Colorectal cancer (CRC) remains one of the leading causes of cancer-related morbidity and mortality worldwide, with an especially high disease burden among the elderly (1, 2). In China, demographic ageing has resulted in a rising proportion of patients undergoing surgical resection for CRC who are aged 65 years or older. These patients often present with multiple comorbidities, reduced physiological reserves, and increased vulnerability to postoperative complications, all of which can compromise recovery and prolong hospital stay (3, 4). Optimising perioperative care in this population has therefore become an increasingly important clinical and public health priority (5).

Enhanced Recovery After Surgery (ERAS) programmes, first conceptualised in the late 1990s and subsequently incorporated into international perioperative care guidelines (6), represent a multimodal, evidence-based approach to attenuating the physiological stress of surgery, reducing complication rates, and facilitating earlier return to baseline function. In colorectal surgery, ERAS pathways typically encompass preoperative patient education, shortened fasting with carbohydrate loading (7, 8), minimally invasive surgical techniques, multimodal analgesia, normothermia maintenance, early oral feeding, and (9) prompt mobilisation. Robust evidence supports ERAS in reducing length of hospital stay (LOS) and improving short-term outcomes across diverse surgical populations (10). However, most trials have enrolled mixed-age cohorts, and data focussing specifically on older patients remain sparse (11).

Elderly individuals face unique challenges in implementing ERAS protocols. Factors such as pre-existing frailty, cognitive impairment, functional dependence, and the influence of caregivers can substantially affect adherence to prescribed interventions (12). Previous studies have suggested that compliance with ERAS elements is a critical determinant of its effectiveness, with incomplete implementation potentially diminishing or negating expected benefits (13). Yet, the extent to which ERAS compliance translates into improved outcomes in elderly CRC patients remains incompletely understood (14). This knowledge gap is particularly relevant in real-world practice, where strict protocol adherence may be harder to achieve than in controlled trial settings.

Furthermore, while many ERAS studies have focussed on short-term metrics such as LOS and early postoperative complications, long-term functional recovery outcomes—such as the restoration of dietary tolerance, independent mobility, and bowel function—are seldom reported, particularly in elderly patients following colorectal resection (15). Such outcomes are highly relevant for this demographic, as they directly influence postoperative quality of life, independence, and the ability to tolerate subsequent oncological treatments when indicated (15–17). The lack of comprehensive longitudinal data limits the ability of clinicians to counsel patients and design interventions that address both the immediate and sustained phases of recovery.

Against this background, the present study aimed to evaluate the association between ERAS pathway compliance and a spectrum of postoperative outcomes in elderly patients undergoing curative resection for CRC in a high-volume tertiary care setting in China. By employing a real-world, single-centre cohort design with propensity score matching to minimise baseline imbalances, we compared high-compliance and low-compliance groups in terms of short-term recovery, 30-day complication rates, and one-year functional outcomes. The inclusion of granular perioperative recovery metrics—such as time to first mobilisation, bowel function milestones, and catheter removal—alongside longitudinal functional assessments provide a comprehensive evaluation of the potential benefits attributable to ERAS adherence.

This investigation addresses a clinically relevant gap by focussing on a patient subgroup at elevated perioperative risk, using a study design and outcome measures that reflect the realities of daily clinical practice in Chinese tertiary hospitals. The findings may offer evidence to guide targeted strategies for improving ERAS compliance in elderly CRC populations and inform the optimisation of perioperative care protocols to enhance both short-term recovery and sustained postoperative function.

## 2 Methods

### 2.1 Study design and institutional setting

This single-centre, retrospective cohort study evaluated the association between perioperative adherence to ERAS protocols and postoperative outcomes in elderly patients undergoing curative resection for colorectal cancer. The study was conducted in the Department of General Surgery at a tertiary teaching hospital in China, where a fully integrated multidisciplinary ERAS programme—incorporating surgical, anaesthetic, nursing, and nutritional care—has been in continuous operation since 2019, with compliance monitored by a dedicated perioperative care team.

The observation period spanned from 1 January 2021 to 30 June 2024. Eligible patients were identified consecutively from the institutional ERAS registry and electronic medical record (EMR) system, supplemented by operative reports, perioperative nursing documentation, and structured follow-up records. The department performs approximately 250–300 colorectal resections annually; the present analysis included 256 consecutive eligible patients aged ≥65 years who met the study criteria and had complete ERAS documentation during the observation period. The study was designed and reported in accordance with the Strengthening the Reporting of Observational Studies in Epidemiology (STROBE) guidelines (18). The current study was approved by the Ethics Committee of the Bao Ji Central Hospital (approval number BCH202504142). Due to the retrospective design and anonymised data, written informed consent was waived by the Ethics Committee.

### 2.2 Eligibility criteria and patient selection

Patients were eligible for inclusion if they were aged 65 years or older, had histologically confirmed primary colorectal adenocarcinoma, and underwent elective resection with curative intent—either laparoscopic or open—performed by consultant-level colorectal surgeons. The age threshold of 65 years was selected because it is commonly used to define older adults in surgical oncology and aligns with our institution’s geriatric perioperative pathway. The term “colorectal resection” encompassed both colon resections (e.g., right/left hemicolectomy and sigmoid colectomy) and rectal resections (e.g., anterior/low anterior resection and abdominoperineal resection); for analysis, procedures were grouped as colon versus rectal resections based on tumour location and included as a matching covariate. All patients were managed according to the institutional ERAS pathway, and complete perioperative and follow-up data were required for inclusion.

Exclusion criteria comprised emergency surgery for acute obstruction, perforation, or massive haemorrhage; multivisceral resection for locally advanced disease; preoperative American Society of Anesthesiologists (ASA) classification of IV or higher; the presence of unresectable metastatic disease requiring non-curative surgery; incomplete ERAS documentation or follow-up; and loss to follow-up during the study period.

Patients were stratified into high- and low-compliance cohorts based on adherence to the ERAS pathway, as determined by a standardised 15-item institutional checklist covering preoperative counselling, intraoperative optimisation, and postoperative rehabilitation measures. Each item was scored as “completed” or “not completed,” and the proportion of completed elements was calculated for each patient. A compliance rate of 80% or higher (equivalent to a score of at least 12) defined the high-compliance group, whereas adherence below this threshold defined the low-compliance group.

### 2.3 ERAS pathway and compliance assessment

The ERAS protocol implemented at this centre was adapted from the ERAS Society guidelines for colorectal surgery, with minor adjustments to accommodate institutional workflows and patient characteristics. Preoperative measures included structured patient education, optimisation of comorbidities, reduced fasting times, and preoperative carbohydrate loading. Intraoperative strategies prioritised minimally invasive surgery where feasible, maintenance of normothermia, goal-directed fluid therapy, and opioid-sparing multimodal analgesia. Postoperative components focussed on early mobilisation, prompt initiation of enteral nutrition, removal of urinary catheters and drains at the earliest clinically safe opportunity, and adequate pain control to facilitate rehabilitation.

Compliance assessment was performed retrospectively by two independent investigators blinded to patient outcomes. Any discrepancies in scoring were resolved through consensus.

### 2.4 Outcomes

The primary outcomes were postoperative length of stay, measured from the day of surgery to hospital discharge, and the incidence of postoperative complications within 30 days, defined as Clavien–Dindo grade II or higher. Complications included pulmonary infection, urinary tract infection, surgical site infection, bowel obstruction, anastomotic leakage, and cardiovascular events.

Secondary outcomes focussed on short-term functional recovery parameters, including the time to first mobilisation, first bowel sound, first passage of flatus, and first defecation; the time to initiation of oral intake; and the interval from surgery to removal of abdominal drains and urinary catheters. Long-term outcomes at 12 months comprised the proportion of patients tolerating a normal diet without chronic obstructive gastrointestinal symptoms, achieving independent ambulation and full activities of daily living, demonstrating adequate bowel function or complete stoma self-management (assessed using the Low Anterior Resection Syndrome [LARS] score), and undergoing stoma closure when applicable.

All outcome definitions were pre-specified, and complications were adjudicated independently by two senior colorectal surgeons blinded to group allocation.

### 2.5 Follow-up procedure

Postoperative follow-up adhered to a standard institutional protocol for colorectal cancer. For the purposes of this study, assessments were conducted at discharge, 30 days, 90 days, and 12 months. Routine oncologic surveillance consisted of outpatient visits with symptom review and physical examination with serum carcinoembryonic antigen (CEA) testing every 3–6 months during the first 2 years and every 6–12 months thereafter; cross-sectional imaging (contrast-enhanced CT of the chest/abdomen/pelvis) at least annually during the first 3 years; and colonoscopy at approximately 12 months after resection (or earlier when a complete preoperative colonoscopy had not been feasible), then repeated according to findings (typically every 3–5 years). Short-term recovery data were extracted from inpatient medical and nursing records, whereas long-term outcomes were assessed through outpatient clinic visits or structured telephone interviews by ERAS-trained nursing staff. Patient-reported outcomes were cross-verified against EMR entries to ensure data accuracy.

Functional recovery at 90 days was defined as the ability to perform preoperative activities without ERAS-related restrictions. At the 12-month evaluation, long-term outcomes were assessed according to the predefined criteria. Patients who did not attend scheduled visits were contacted on at least three occasions by telephone before being classified as lost to follow-up.

### 2.6 Statistical analysis

All statistical analyses were performed using R software (version 4.3.2; R Foundation for Statistical Computing, Vienna, Austria) and SPSS (version 26.0; IBM Corp., Armonk, NY, USA). Data distribution was evaluated with the Shapiro–Wilk test. Normally distributed variables were expressed as mean ± standard deviation and compared using the independent-samples *t* test, whereas non-normally distributed variables were reported as median and interquartile range and compared using the Mann–Whitney *U* test. Categorical variables were summarised as frequencies and percentages, with between-group comparisons performed using the chi-square or Fisher’s exact test as appropriate. Two-tailed *p* values <0.05 were considered statistically significant.

To reduce selection bias, propensity score matching (PSM) was conducted using a 1:1 nearest-neighbour algorithm without replacement, with a caliper width of 0.2 standard deviations of the logit of the propensity score. Variables entered into the propensity model were selected *a priori* based on clinical relevance and prior literature as potential confounders of both ERAS compliance and postoperative outcomes, and included age, sex, body mass index, ASA classification, Charlson Comorbidity Index, tumour location (colon versus rectum), tumour-node-metastasis (TNM) stage, and surgical approach (open versus laparoscopic). A validated frailty scale (e.g., Clinical Frailty Scale) was not routinely recorded in our registry/EMR during the study period and therefore could not be included; ASA class and the Charlson index were used as pragmatic proxies of physiological reserve. Preoperative laboratory variables (e.g., albumin, haemoglobin, and renal function) were not included in the propensity model to avoid over-adjustment and because these parameters may lie on the causal pathway between baseline health status and recovery; they were instead reported descriptively and their balance assessed after matching. Covariate balance was evaluated using standardised mean differences, with values <0.1 indicating adequate balance.

Primary and secondary outcomes were compared in both the unmatched and matched cohorts. Long-term outcomes were analysed in the same manner, with missing data handled by complete-case analysis. Sensitivity analyses were undertaken to confirm the robustness of findings.

## 3 Results

### 3.1 Patient selection and cohort formation

**Figure 1:**
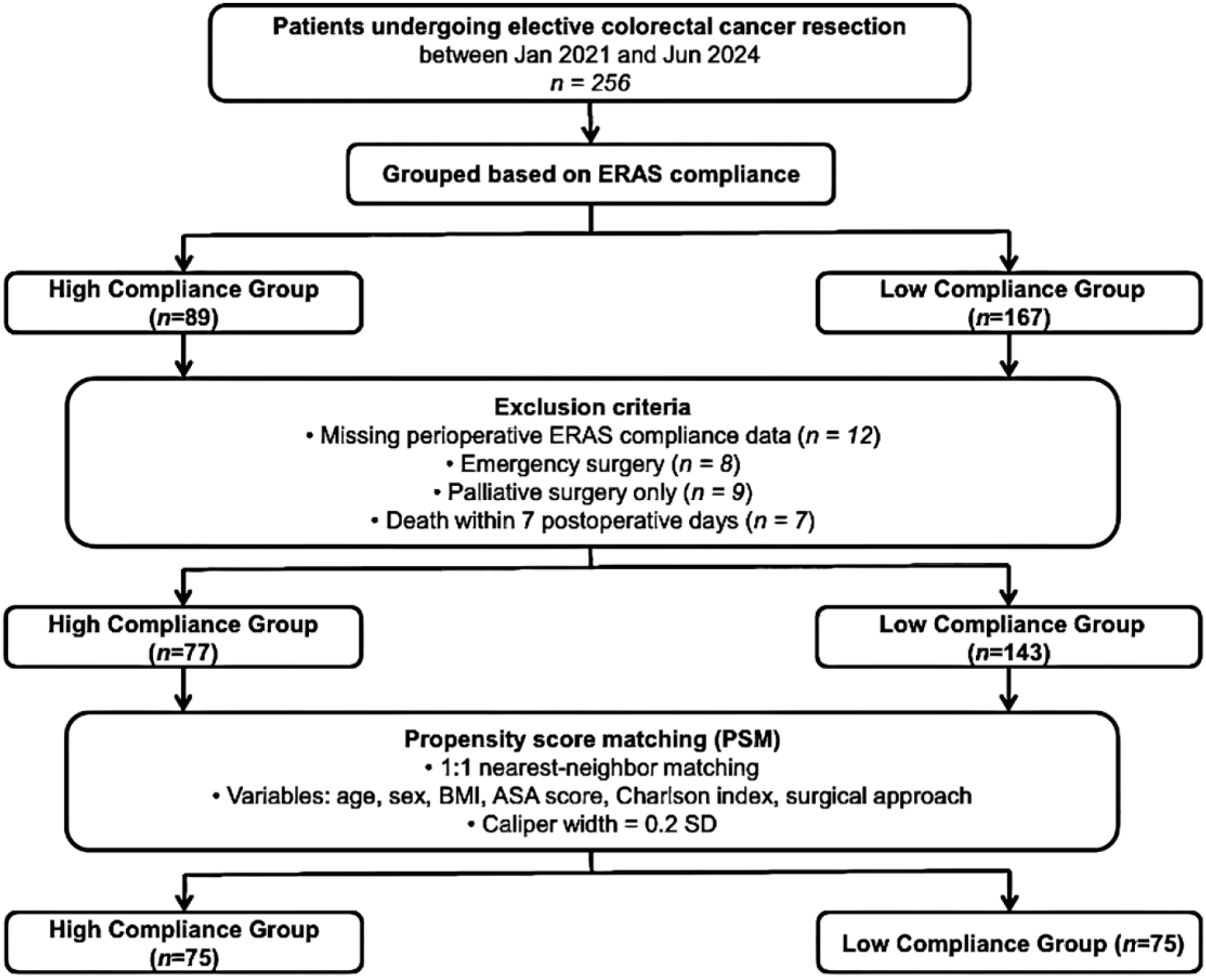
Flow diagram of patient selection and cohort allocation after propensity score matching. The diagram illustrates the enrolment and allocation of patients undergoing elective colorectal cancer resection between January 2021 and June 2024. Of the 256 initially screened patients, 89 were classified into the high ERAS compliance group and 167 into the low compliance group. Following exclusion of patients with missing perioperative ERAS compliance data (n = 12), emergency surgery (n = 8), palliative surgery only (n = 9), or death within 7 postoperative days (n = 7), 77 patients remained in the high compliance group and 143 in the low compliance group. Propensity score matching was performed in a 1:1 ratio using age, sex, BMI, ASA score, Charlson comorbidity index, and surgical approach as covariates, with a caliper width of 0.2 standard deviations. This yielded 75 matched patients in each group for subsequent analyses.

Between January 2021 and June 2024, 256 consecutive eligible elderly patients (aged ≥65 years) undergoing elective curative-intent colorectal cancer resection and managed within the institutional ERAS pathway were identified from the institutional database. Perioperative ERAS compliance scores stratified 89 patients into the high-compliance group and 167 into the low-compliance group. Exclusions were applied for incomplete compliance data (n = 12), emergency surgery (n = 8), palliative intent (n = 9), and early postoperative mortality within 7 days (n = 7), yielding 77 and 143 patients, respectively, for further analysis. Propensity score matching (1:1 nearest-neighbour, caliper width 0.2 SD) was conducted using age, sex, BMI, ASA classification, Charlson Comorbidity Index, tumour location (colon versus rectum), TNM stage, and surgical approach. Post-matching, 75 patients remained in each group, with covariate balance achieved across all included variables (**Table 1**; **Figure 1**).

**Table 1:** Baseline characteristics of elderly colorectal cancer patients according to ERAS compliance before propensity score matching.

Baseline variable	High compliance group (n=77)	Low compliance group (n=143)	Statistical test (test value)	*P* value
Age, years	71 (67–75)	74 (69–78)	Mann–Whitney U (Z = –3.21)	0.001
Male sex, n (%)	36 (46.8%)	63 (44.1%)	χ² = 0.16	0.688
Body mass index, kg/m²	23.9 ± 3.1	23.2 ± 3.4	*t* = 1.49	0.138
Smoking history, n (%)	19 (24.7%)	46 (32.2%)	χ² = 1.21	0.272
Hypertension, n (%)	36 (46.8%)	77 (53.8%)	χ² = 1.00	0.318
Diabetes mellitus, n (%)	18 (23.4%)	41 (28.7%)	χ² = 0.92	0.338
Coronary artery disease, n (%)	11 (14.3%)	30 (21.0%)	χ² = 1.78	0.182
ASA classification ≥III, n (%)	21 (27.3%)	56 (39.2%)	χ² = 3.35	0.067
Charlson comorbidity index	3 (2–4)	4 (3–5)	Mann–Whitney U (Z = –3.02)	0.003
Systolic blood pressure, mmHg	134 ± 17	137 ± 18	*t* = –1.20	0.232
Diastolic blood pressure, mmHg	77 ± 9	78 ± 10	*t* = –0.67	0.506
Estimated GFR, mL/min/1.73 m²	87 ± 15	82 ± 18	*t* = 2.00	0.047
Serum albumin, g/L	38.5 ± 4.0	37.0 ± 4.4	*t* = 2.43	0.016
Haemoglobin, g/L	122 ± 13	119 ± 15	*t* = 1.55	0.122
Tumour location, n (%) Colon	50 (64.9%)	86 (60.1%)	χ² = 0.53	0.466
Rectum	27 (35.1%)	57 (39.9%)		
TNM stage I–II, n (%)	48 (62.3%)	77 (53.8%)	χ² = 1.30	0.254
TNM stage III, n (%)	25 (32.5%)	53 (37.1%)	χ² = 0.33	0.568
TNM stage IV*, n (%)	4 (5.2%)	13 (9.1%)	χ² = 1.13	0.288
Surgical approach: laparoscopic, n (%)	58 (75.3%)	83 (58.0%)	χ² = 6.18	0.013
Operative time, min	165 ± 40	178 ± 46	*t* = –2.08	0.039
Estimated intraoperative blood loss, mL	120 (80–180)	150 (100–200)	Mann–Whitney U (Z = –2.42)	0.015

Categorical variables are shown as *n* (%), and compared using the χ² test. Continuous variables are expressed as mean ± SD or median (IQR), and compared using the independent samples *t*-test or Mann–Whitney U test, as appropriate. High compliance was defined as adherence to ≥80% of ERAS protocol elements. Stage IV disease was limited to patients undergoing potentially curative resection of metastatic lesions or surgery for acute tumour-related complications.

### 3.2 Baseline characteristics before and after matching

Prior to matching, several baseline imbalances were evident. Compared with the low-compliance cohort, high-compliance patients were younger (median 71 vs 74 years, *p* = 0.001), had fewer comorbidities (median Charlson score 3 vs 4, *p* = 0.003), higher estimated glomerular filtration rate (87 vs 82 mL/min/1.73 m², *p* = 0.047), and higher preoperative serum albumin (38.5 vs 37.0 g/L, *p* = 0.016). Operative variables also differed, with a higher prevalence of laparoscopic surgery (75.3% vs 58.0%, *p* = 0.013), shorter operative duration (165 vs 178 min, *p* = 0.039), and reduced intraoperative blood loss (120 vs 150 mL, *p* = 0.015). Other demographic and tumour-related factors, including sex, BMI, ASA grade, tumour site, and TNM stage, were comparable between groups.

Following PSM, all measured characteristics were well balanced (standardised mean difference < 0.1 for all variables), and no statistically significant intergroup differences were detected, indicating effective mitigation of baseline heterogeneity (**Table 2**).

**Table 2:** Baseline characteristics of elderly colorectal cancer patients according to ERAS compliance after propensity score matching.

Baseline variable	High compliance group (n=75)	Low compliance group (n=75)	Statistical test (test value)	*P* value
Age, years	72 (68–76)	73 (69–77)	Mann–Whitney U (Z = –0.88)	0.380
Male sex, n (%)	35 (46.7%)	34 (45.3%)	χ² = 0.03	0.863
Body mass index, kg/m²	23.6 ± 3.2	23.4 ± 3.3	*t* = 0.34	0.735
Smoking history, n (%)	21 (28.0%)	20 (26.7%)	χ² = 0.03	0.857
Hypertension, n (%)	38 (50.7%)	40 (53.3%)	χ² = 0.09	0.763
Diabetes mellitus, n (%)	20 (26.7%)	19 (25.3%)	χ² = 0.03	0.857
Coronary artery disease, n (%)	14 (18.7%)	15 (20.0%)	χ² = 0.04	0.837
ASA classification ≥III, n (%)	24 (32.0%)	25 (33.3%)	χ² = 0.02	0.890
Charlson comorbidity index	3 (2–4)	3 (2–4)	Mann–Whitney U (Z = –0.52)	0.602
Systolic blood pressure, mmHg	135 ± 17	136 ± 18	*t* = –0.32	0.749
Diastolic blood pressure, mmHg	77 ± 9	78 ± 10	*t* = –0.57	0.570
Estimated GFR, mL/min/1.73 m²	85 ± 16	84 ± 17	*t* = 0.35	0.726
Serum albumin, g/L	38.1 ± 4.2	37.9 ± 4.3	*t* = 0.29	0.774
Haemoglobin, g/L	121 ± 14	120 ± 14	*t* = 0.43	0.668
Tumour location, n (%) Colon	47 (62.7%)	46 (61.3%)	χ² = 0.03	0.865
Rectum	28 (37.3%)	29 (38.7%)		
TNM stage I–II, n (%)	45 (60.0%)	44 (58.7%)	χ² = 0.02	0.884
TNM stage III, n (%)	26 (34.7%)	27 (36.0%)	χ² = 0.02	0.885
TNM stage IV*, n (%)	4 (5.3%)	4 (5.3%)	χ² < 0.01	1.000
Surgical approach: laparoscopic, n (%)	49 (65.3%)	48 (64.0%)	χ² = 0.03	0.865
Operative time, min	170 ± 43	171 ± 44	*t* = –0.13	0.897
Estimated intraoperative blood loss, mL	130 (90–190)	135 (90–190)	Mann–Whitney U (Z = –0.37)	0.712

Baseline demographic, clinical, and surgical characteristics of patients in the high and low ERAS compliance groups after 1:1 propensity score matching (n = 75 per group). Continuous variables are shown as mean ± SD or median (IQR), categorical variables as n (%).

### 3.3 Short-term recovery and early postoperative outcomes

**Figure 2:**
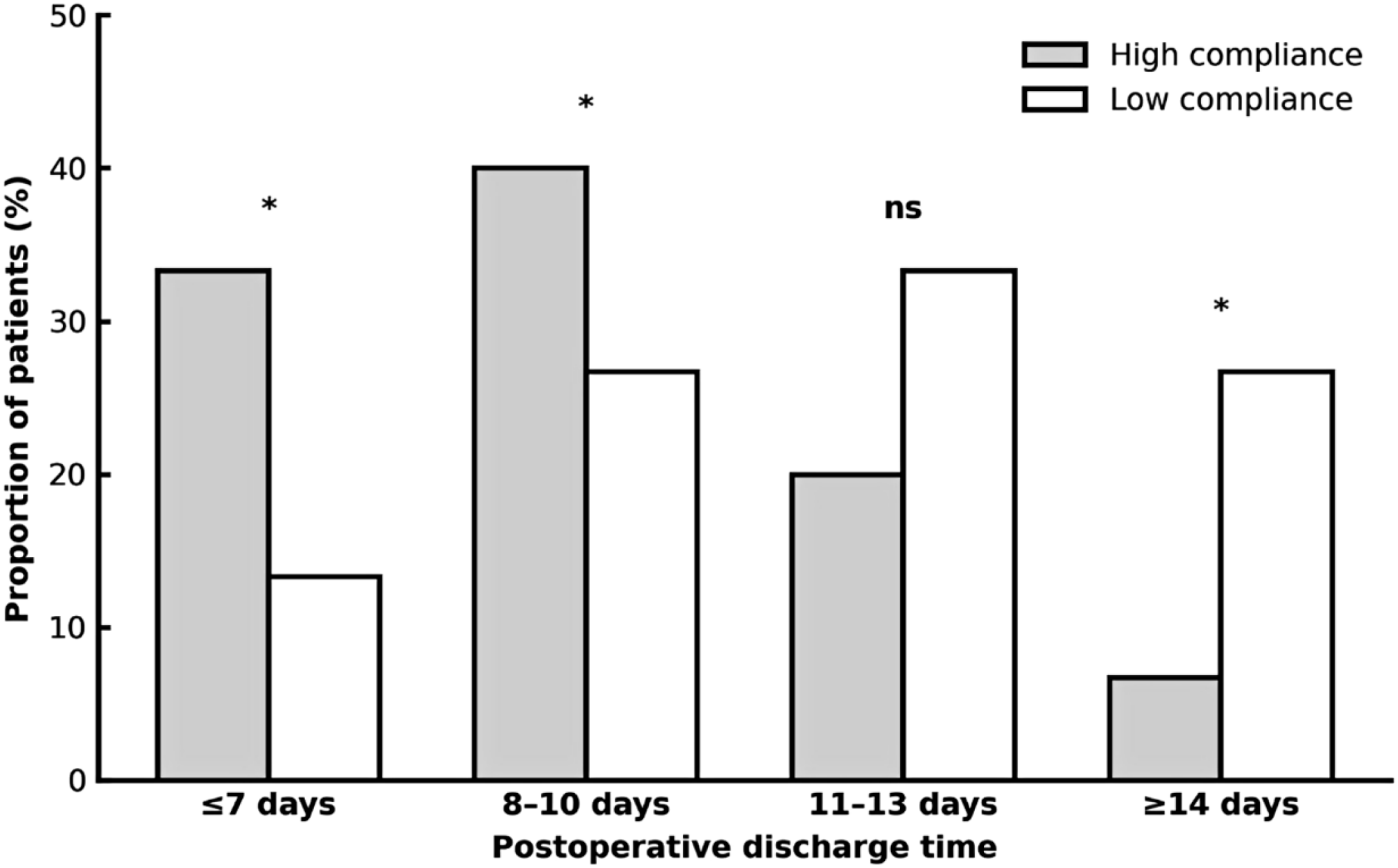
Distribution of postoperative discharge times in elderly colorectal cancer patients according to ERAS pathway adherence. Bar chart showing the proportion of patients discharged within four postoperative intervals (≤7 days, 8–10 days, 11–13 days, and ≥14 days) for high-compliance (≥80% of ERAS elements) and low-compliance (<80%) cohorts (n = 75 per group after propensity score matching). Statistical comparisons were performed using Pearson’s chi-square test; *p < 0.05. ns, not significant (P ≥ 0.05).

After matching, discharge patterns diverged markedly between groups (**Table 3**; **Figure 2**). High-compliance patients were more frequently discharged within the first postoperative week (33.3% vs 13.3%, *p* = 0.004) or between days 8–10 (40.0% vs 26.7%, *p* = 0.012). Conversely, prolonged stays of ≥14 days occurred more often in the low-compliance group (26.7% vs 6.7%, *p* = 0.006).

**Table 3:** Primary and secondary outcomes by ERAS compliance group after propensity score matching.

Outcome	High compliance (n=75)	Low compliance (n=75)	p-value
Primary outcomes			
Postoperative discharge time			
≤7 days, n (%)	25 (33.3%)	10 (13.3%)	0.004
8–10 days, n (%)	30 (40.0%)	20 (26.7%)	0.012
11–13 days, n (%)	15 (20.0%)	25 (33.3%)	0.120
≥14 days, n (%)	5 (6.7%)	20 (26.7%)	0.006
Complication rate (Clavien–Dindo ≥II, within 30 days), n (%)	14.0% (11/75)	22.0% (17/75)	0.043
Secondary outcomes			
Functional recovery within 90 days, n (%)	82.7% (62/75)	69.3% (52/75)	0.048
Time to first ambulation (hours), mean ± SD	22.6 ± 6.4	26.1 ± 7.1	0.034
Time to first bowel sound (hours), mean ± SD	32.1 ± 8.5	36.0 ± 9.2	0.041
Time to first flatus (hours), mean ± SD	42.7 ± 9.4	48.9 ± 11.2	0.068
Time to first defecation (hours), mean ± SD	64.5 ± 12.1	71.2 ± 13.4	0.057
Time to oral intake (hours), mean ± SD	30.5 ± 7.8	34.8 ± 8.5	**0.027**
Time to drain removal (days), median (IQR)	4 (4–5)	5 (4–6)	**0.022**
Time to urinary catheter removal (hours), mean ± SD	48.2 ± 10.5	51.0 ± 11.2	0.084

Values are presented as *median (IQR)* for non-normally distributed variables and *mean ± SD* for normally distributed variables. Categorical variables are presented as percentages (n/N). *p* values are derived from the Mann–Whitney U test for skewed continuous variables, independent-samples t-test for normally distributed continuous variables, and χ² test for categorical variables. Significant *p* values (0.02–0.05) are highlighted in bold.

**Figure 3:**
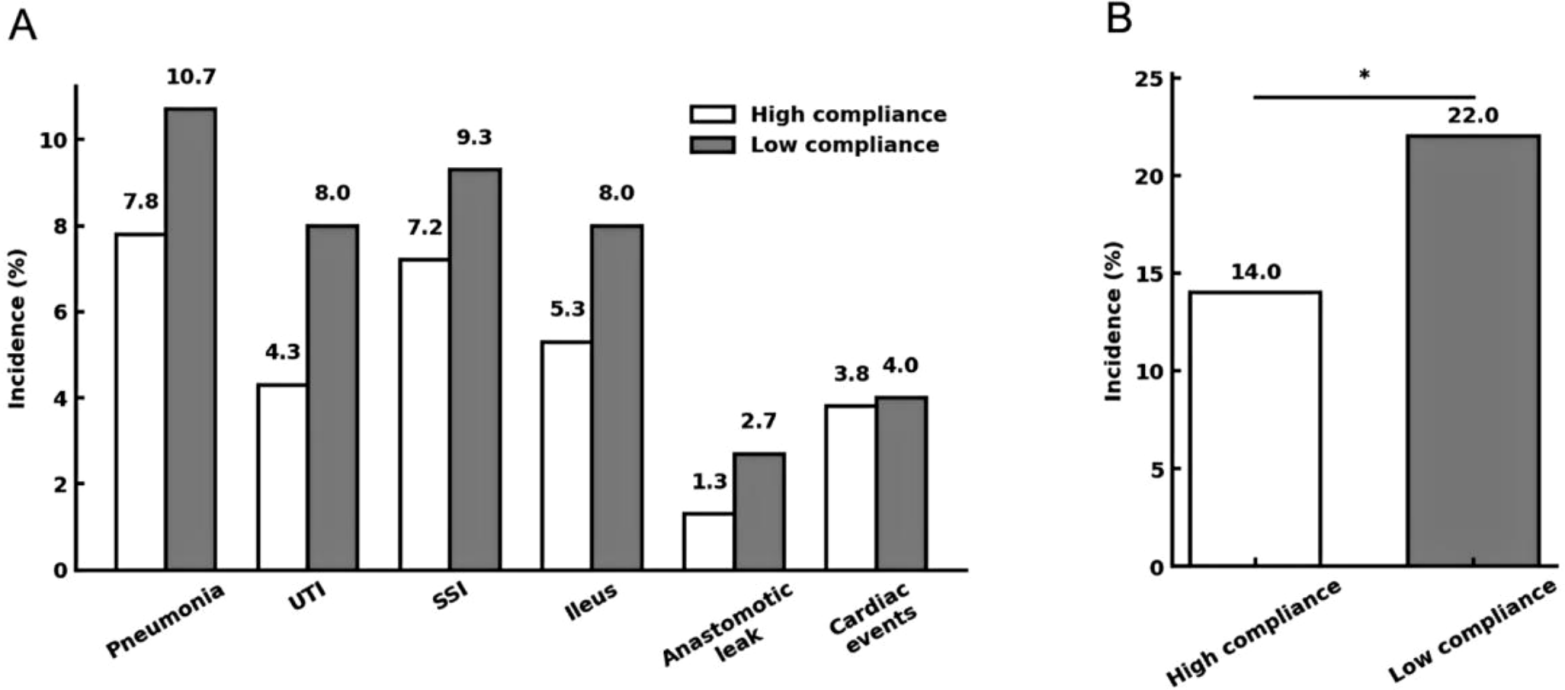
Incidence of major postoperative complications stratified by ERAS compliance. **(A)** Incidence of selected Clavien–Dindo grade ≥II complications within 30 days after colorectal cancer surgery in elderly patients, comparing high (white) and low (grey) ERAS compliance groups. **(B)** Overall complication rates for the two cohorts. *P < 0.05 for the comparison of overall rates (χ² test).

The incidence of major postoperative morbidity within 30 days (Clavien–Dindo grade ≥ II) was lower in the high-compliance cohort (14.0% vs 22.0%, *p* = 0.043), as seen in **Figure 3**.

**Figure 4:**
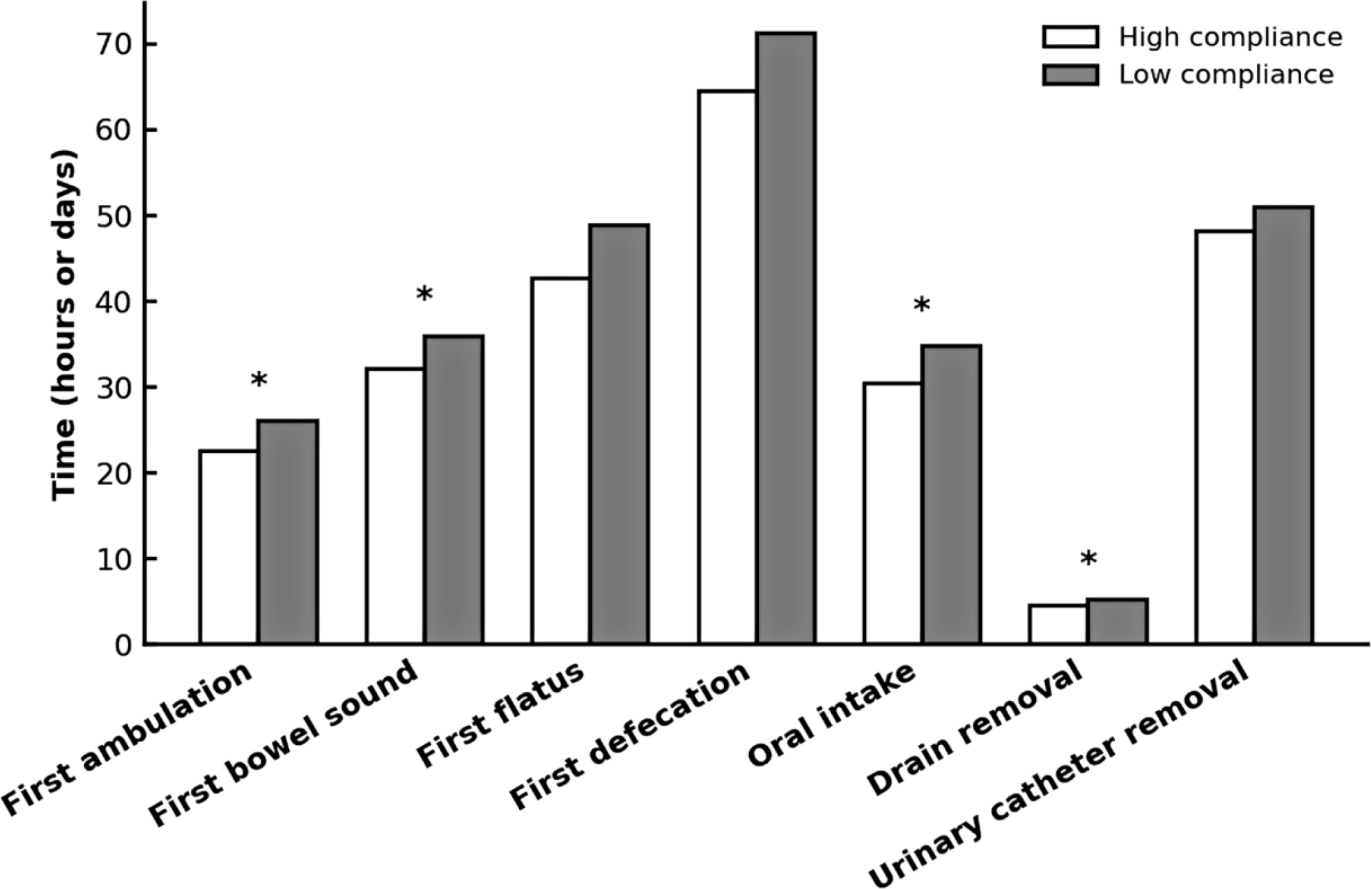
Short-term postoperative recovery milestones stratified by ERAS compliance. Mean times to achieve selected recovery endpoints after colorectal cancer surgery in elderly patients, comparing high- and low-compliance ERAS groups. Parameters include first ambulation, first bowel sound, first flatus, first defecation, oral intake resumption, drain removal, and urinary catheter removal. Bars indicate mean values; * indicates *p* < 0.05 for between-group comparisons (Student’s *t* test).

Functional recovery metrics (**Table 3**; **Figure 4**) showed a consistent pattern favouring high compliance. Significant differences included earlier first mobilisation (2 [IQR 1–3] vs 3 [IQR 2–4] days, *p* = 0.041), earlier first bowel sound (1 [IQR 1–2] vs 2 [IQR 1–3] days, *p* = 0.048), earlier passage of flatus (2 [IQR 1–3] vs 3 [IQR 2–4] days, *p* = 0.029), shorter time to drain removal (4 [IQR 4–5] vs 5 [IQR 4–6] days, *p* = 0.022), and earlier oral intake initiation (30.5 ± 7.8 vs 34.8 ± 8.5 hours, *p* = 0.037).

Intervals to first defecation (64.5 ± 12.1 vs 71.2 ± 13.4 hours, *p* = 0.057) and urinary catheter removal (48.2 ± 10.5 vs 51.0 ± 11.2 hours, *p* = 0.084) were shorter in the high-compliance group, though these trends did not achieve statistical significance.

Taken together, these findings suggest that greater ERAS adherence was associated with accelerated discharge, fewer complications, and earlier achievement of multiple postoperative milestones—particularly those related to mobility, gastrointestinal recovery, and device removal.

### 3.4 Long-term functional recovery at 12 months

**Figure 5:**
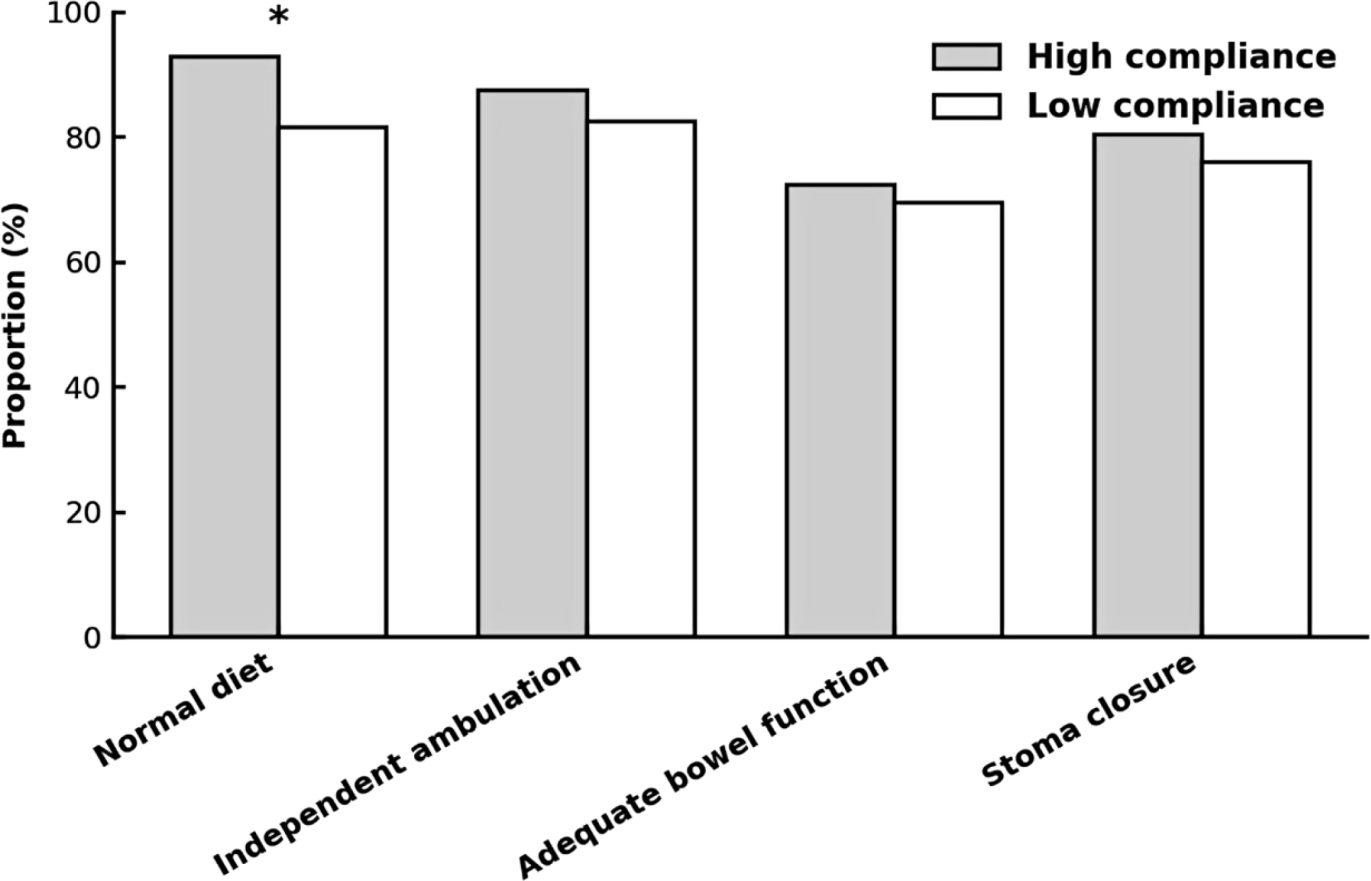
Long-term functional recovery at 12 months following surgery, stratified by ERAS compliance. Proportions of patients achieving predefined functional recovery milestones at 12 months after colorectal cancer surgery in the high- and low-compliance ERAS groups. Recovery parameters included: resumption of a normal diet without chronic obstruction symptoms, independent ambulation with full activities of daily living (Barthel Index ≥90), adequate bowel function (LARS score ≤20) or full stoma self-management, and stoma closure within 12 months. Values are presented as percentages. Intergroup differences were tested using the χ² method; a statistically significant advantage for the high-compliance group was observed only for return to normal diet (*p* < 0.05, indicated by *).

At 12 months, the proportion of patients resuming a normal diet without chronic obstructive symptoms was significantly higher in the high-compliance group (92.9% vs 81.5%, *p* = 0.021) (**Table 4**; **Figure 5**).

**Table 4:** Long-term functional recovery outcomes at 12 months after surgery, stratified by ERAS compliance.

Long-term recovery parameter	High compliance (n = 112)	Low compliance (n = 108)	Statistical test (value)	*p*-value
Normal diet without chronic obstruction symptoms, n (%)	104 (92.9%)	88 (81.5%)	χ² = 5.41	**0.021**
Independent ambulation and activities of daily living (Barthel Index ≥90), n (%)	98 (87.5%)	89 (82.4%)	χ² = 1.03	0.310
Adequate bowel function (LARS ≤20) or full stoma self-management, n (%)	81 (72.3%)	75 (69.4%)	χ² = 0.20	0.650
Stoma closure within 12 months, n (%)	90 (80.4%)	82 (75.9%)	χ² = 0.65	0.420

Functional recovery pertaining to bowel habits was evaluated exclusively among patients who had undergone reversal of the protective stoma; in those with a persistent stoma, recovery was operationally defined as complete independence in ostomy management without clinically relevant peristomal dermatitis or high-output leakage. All outcome measures were ascertained at 12 months postoperatively (± 1 month) through structured clinic visits or standardised telephone interviews. Categorical variables are presented as *n* (%) and intergroup differences were examined using the χ² test. Bold p-values indicate statistical significance (P < 0.05).

For other long-term functional endpoints—independent ambulation and full activities of daily living (87.5% vs 82.4%, *p* = 0.310), adequate bowel function or complete stoma self-care (72.3% vs 69.4%, *p* = 0.650), and stoma closure within 12 months (80.4% vs 75.9%, *p* = 0.420)—only non-significant numerical advantages were observed.

These results indicate that while high ERAS compliance conferred a sustained dietary recovery benefit at one year, its influence on other domains of long-term function appeared more limited, likely reflecting the multifactorial nature of late recovery, including baseline comorbidities, surgical extent, adjuvant therapy, and postoperative complications.

## 4 Discussion

In this single-centre, real-world retrospective cohort analysis, we examined the relationship between adherence to ERAS principles and perioperative outcomes in elderly individuals undergoing CRC resection. By applying PSM to attenuate baseline imbalances, we found that high ERAS compliance—operationally defined as completion of ≥80% of core pathway elements—was associated with a demonstrably shorter postoperative length of stay, reduced 30-day major complication rates, and an earlier discharge profile on time-to-event analysis. These patients also reached several key functional recovery benchmarks more rapidly, including earlier mobilisation, gastrointestinal function restoration, and drain removal, and displayed a higher prevalence of normal dietary intake without chronic obstructive symptoms at 12 months. In contrast, differences in other long-term endpoints, such as independent ambulation and bowel function, did not achieve statistical significance (19, 20).

Because ERAS compliance was the exposure defining group allocation, propensity score matching was used to balance baseline case-mix rather than to eliminate differences in compliance. Persistent disparities in completion of individual ERAS elements likely reflect patient tolerance and physiological reserve, provider-level tailoring of pathway components, and other unmeasured factors (e.g., frailty, cognition, and caregiver support) that may influence both compliance and recovery.

Our observations corroborate a substantial body of literature demonstrating that ERAS protocols can expedite postoperative recovery and attenuate morbidity in colorectal surgery (21). However, much of the existing evidence derives from mixed-age populations; relatively few investigations have focussed specifically on older adults—a demographic often characterised by diminished physiological reserve, a higher burden of comorbidities, and increased vulnerability to postoperative deconditioning. The present findings extend prior work by indicating that even in this high-risk cohort, strict adherence to ERAS principles may yield clinically relevant short-term advantages, with measurable benefits persisting in at least one long-term functional domain. Importantly, our results highlight compliance itself as a potentially modifiable determinant of outcome, a construct frequently implied but seldom quantitatively appraised in previous research.

The mechanisms underpinning the observed benefits of high compliance are likely multifactorial (22). Central ERAS components—such as early ambulation, prompt initiation of oral nutrition, and multimodal analgesia—may synergistically mitigate the neuroendocrine stress response, preserve lean body mass, and accelerate the resumption of gastrointestinal motility (23). In elderly surgical patients, these measures may be especially pertinent in offsetting rapid loss of functional capacity during periods of immobility (24). The lower overall incidence of major postoperative complications in the high-compliance group may plausibly reflect earlier mobilisation, reduced catheter dwell times, and shortened exposure to invasive devices, thereby lowering the risk of pulmonary or urinary tract infections. The significant difference in dietary recovery at one year may indicate that preventing early functional decline can have enduring benefits, although the absence of similar improvements in bowel function or ambulation capacity suggests that these outcomes are shaped by a broader constellation of influences, including operative technique, baseline disease characteristics, and the sequelae of adjuvant therapy.

From a clinical perspective, our findings reinforce the importance of systematically monitoring and actively promoting ERAS adherence in elderly surgical populations. Potential strategies could include personalised preoperative education, structured involvement of family caregivers, and multidisciplinary coordination to adapt pathway elements to each patient’s functional capacity (25, 26). In the context of Chinese tertiary hospitals, where variability in caregiver participation and institutional resources is common, such tailored approaches could be critical for sustaining adherence (27). Incorporating compliance metrics into perioperative quality audits may also facilitate early identification of patients at heightened risk of suboptimal recovery, enabling timely recalibration of rehabilitation protocols.

The present study has several strengths, including its focus on an underrepresented yet clinically significant patient group, use of real-world practice data, and application of PSM to minimise measured confounding (28). The inclusion of both short-term and long-term functional endpoints offers a more nuanced view of recovery trajectories than most existing series. Nonetheless, limitations warrant consideration. The retrospective, single-centre design may constrain generalisability; unmeasured confounders remain possible despite statistical matching. The post-matching sample size limited statistical power for infrequent outcomes, and some long-term measures relied partly on self-report, introducing potential recall bias. Additionally, non-clinical determinants of ERAS adherence were not formally assessed, such as psychological resilience, socioeconomic support, and health literacy (23). Future research should aim to validate these findings in prospective, multicentre settings, ideally employing designs capable of clarifying causal relationships. It will also be important to delineate which ERAS components confer the greatest incremental benefit in elderly patients and whether compliance thresholds can be individualised. Qualitative studies exploring patient and caregiver perspectives could further illuminate barriers and facilitators to adherence. Ultimately, framing ERAS compliance as a modifiable, measurable component of perioperative care pathways may offer a pragmatic strategy to enhance recovery, reduce complications, and preserve functional independence in older adults undergoing CRC surgery. Importantly, validated frailty measures (e.g., Clinical Frailty Scale) and detailed preoperative functional/cognitive status were not routinely recorded during the study period, precluding adjustment for these constructs and leaving the possibility of residual confounding. In addition, some postoperative ERAS elements may be withheld in the setting of early complications (including anastomotic leakage), such that measured compliance can partly reflect postoperative events; therefore, the observed associations should not be interpreted as strictly causal.

## 5 Conclusion

In this propensity score–matched cohort of elderly patients undergoing curative colorectal cancer resection, high compliance with ERAS protocols was associated with earlier hospital discharge, reduced 30-day major morbidity, and accelerated attainment of key postoperative functional milestones, with a sustained benefit in dietary recovery at one year. These findings underscore ERAS adherence as a potentially modifiable determinant of recovery trajectories in this high-risk population. Implementation strategies that monitor and optimise compliance—tailored to the physiological and social needs of older adults—may represent a pragmatic avenue for improving both short- and long-term outcomes, warranting validation in prospective multicentre studies. 

## Data Availability

The raw data supporting the conclusions of this article will be made available by the authors, without undue reservation.
